# Synthesis and Characterization of Long-Chain Tartaric Acid Diamides as Novel Ceramide-Like Compounds

**DOI:** 10.3390/molecules15020824

**Published:** 2010-02-05

**Authors:** Bálint Sinkó, Melinda Pálfi, Szabolcs Béni, József Kökösi, Krisztina Takács-Novák

**Affiliations:** 1 Department of Pharmaceutical Chemistry, Semmelweis University, Hőgyes E.u.9., Budapest H-1092, Hungary; E-Mails: sinbal@gytk.sote.hu (B.S.); beniszabi@gytk.sote.hu (S.B.); kozsef@gytk.sote.hu (J.K.); 2 Department of Pharmacodynamics, Semmelweis University, Nagyvárad tér 4., Budapest H-1089, Hungary; E-Mail: melinda.palfi@net.sote.hu (M.P.)

**Keywords:** ceramide-analogues, tartradiamides, transdermal penetration, apoptosis

## Abstract

Ceramides play a crucial role in the barrier function of the skin as well as in transmembrane signaling. In this study long aliphatic chain tartaric acid diamides able to replace ceramides in an *in vitro* model of the *stratum corneum* lipid matrix due to their similar physico-chemical properties were synthesized from diacetoxysuccinic anhydride in four steps. Their pro-apoptotic effect on fibroblast cells was also investigated.

## 1. Introduction

The skin acts as a major target as well as a principle barrier for transdermal drug delivery [1]. The *stratum corneum* plays a crucial role in the barrier function for topical drug penetration. Numerous methods have been developed to predict the penetration through the *stratum corneum*, but all these methods suffer several limitations [2,3]. There is a major need for a quick and effective model in early stages of drug discovery, because the possibility of applying drugs transdermally offers fundamental advantages compared to other routes of administration. The Parallel Artificial Membrane Permeability Assay (PAMPA) [4] can be a good method for this purpose provided it applies artificial membrane similar to *stratum corneum*.

As main barrier of the skin, the *stratum corneum* lipid matrix contains as the predominant components about 50% of ceramides, 35% of free fatty acids and 15% of cholesterol; its behavior is different from other biological membranes. Ceramide (*N*-fatty acyl sphingosine) is the lipid moiety of sphingomyelin and all types of glycosphingolipids. In human *stratum corneum* eight subclasses of ceramides have been identified, which differ from each other by the head group architecture and the alkyl chain lengths [5]. The alkyl chain lengths distribution is bimodal, the most abundant chain length is C_24_-C_26_, but there is a small fraction of ceramides having an acyl chain length of C_16_-C_18_ mainly responsible for penetration properties. Recently, the function of ceramide as second messenger in transmembrane signaling has been the subject of great interest [6,7]. They play an important role in regulating the transmission mechanisms of the signals controlled by the membrane sphingolipids [8].

**Figure 1:**
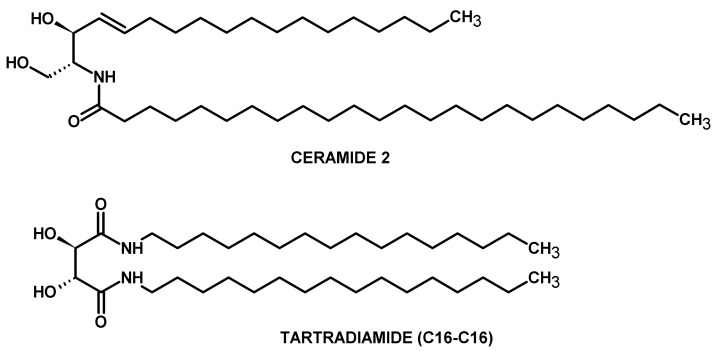
The structure of ceramide 2 and tartramide (C_16_-C_16_).

The endogenous ceramides used in cosmetics are expensive natural extracts, they are mixtures in which the *N*-acyl chain is saturated or unsaturated and may contain a hydroxyl substituent in the alpha position relative to the carbonyl group. In order to replace the endogenous ceramides in artificial membrane of skin PAMPA model, our aim was to develop synthetic ceramide analogues that are able to mimic their effects, are stable enough against metabolization and cheap enough for HT method. Tartaric acid serves as a good basis for the synthesis of ceramides-analogues as its 2,3-diol subunit is able to form hydrogen bonds similarly to real ceramides (Figure 1).

Natural L-(+)-tartaric acid is one of the cheapest enantiomerically pure organic compounds available. It is a polar, polyfunctional and chiral molecule with the absolute configuration of (2*R*,3*R*), and with a *C*2 axis of symmetry. Tartaric acid derivatives have been the subject of numerous studies and display selective ion complexation or ion transport [9], chiral recognition [10] and molecular catalysis [11]. Since tartaric acid esters are hydrolytically too labile [12], the aim was to synthesize long chain tartaric acid amides (tartramides). A 10 member series of ceramide analogues was designed, where the polar portion of ceramide has been replaced by an inverse bioisosteric amide and the lipophilic portion of the C_18_ alkyl moiety of ceramide has been replaced with various long chain (8–18) alkylamines. Stereochemically, both ceramides and tartramides contain two chiral carbon centers and form four stereoisomers: two enantiomeric pairs of erythro and threo-diastereomers.

## 2. Results and Discussion

### 2.1. Synthesis of tartaric acid diamides

In the literature several synthetic routes are known for preparation of tartramides, including pyrolysis of alkylammonium tartarate [10], microwave condensation of tartaric acid with different amines [13,14], and amidation of tartaric acid esters or OH-protected tartaric dichloride [11]. For the synthesis of nonsymmetrical tartramides a multistep reaction route was developed [15]. After formation of tartaric acid monoamide it was reacted with a dehydrating agent to obtain *N*-alkyltartrimide by ring closure, then a ring opening reaction with another aliphatic amide lead to the nonsymmetrical tartramide. The cited synthetic routes have been developed for the preparation of *short* alkyl chain derivatives, as the *long* chain amines (>12) failed to react with tartrimide, even after longer heating at reflux temperature. The aim of this work was to develop a route which is suitable for the synthesis of long alkyl chain tartradiamides.

**Scheme 1:**
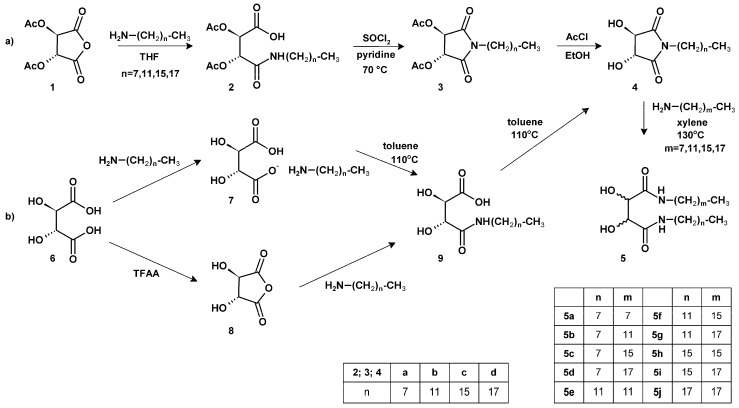
Synthesis of tartaric acid diamides **5.**

Formation of cyclic tartrimide **4** from tartaric acid **6** via tartaric anhydride **8** using trifluoroacetic anhydride lead to formation of *O*-acetylated side product and ring opened diamide. Prolonged thermal heating of the monoammonium salt **7** in toluene under azeotropic reflux also provided various amounts of amide product **9**, depending on the chain length (Scheme 1/b). The products are difficult to purify, which causes diamide mixture formation in the last step. These methods are only useful in small scale. The scale up of these reactions greatly increases the quantity of undesired side products. After reinvestigation of literature methods we have found that a convenient access to the desired products necessitates multistep approach using O-protected tartaric anhydride.

Compounds **5a**–**j** were prepared as shown in Scheme 1/a. Treatment of (*R,R*)-diacetoxysuccinic anhydride (**1**) with the appropriate long-chain amines in THF at room temperature overnight lead to diacetoxysuccinamides **2a**–**d** in high yield. Small amounts of ammonium salt of products could be isolated by filtration, which were crystallized from the reaction mixture. Ring closure of compounds **2a**–**d** with excess thionyl chloride in the presence of catalytic amount of pyridine at 70 °C for 15 min provided the alkyltartrimide derivatives **3a**–**d** as thick semisolid compounds, which were used directly in the next step. Deacetylation of compounds **3a**–**d** with acetyl chloride/ethanol mixture afforded the appropriate tartrimide derivatives **4a**–**d** in good yield. The products were crystallized from the reaction mixtures and isolated by filtration. The reaction of compounds **4a**–**d** with excess of amines in xylene at 130 °C for 24 h yields the tartramides **5a**–**j**. Amine catalyzed racemisation was observed during these reactions, depending on the chain length. The isomer ratio of the products was determined by NMR spectroscopy. Eight of the ten tartramides (**5b, 5c, 5d, 5f, 5g, 5h, 5i, 5j**) are new chemical entities.

### 2.2. Characterization of tartaric acid diamides

The ^1^H- and ^13^C-NMR spectra of chiral tartaric acid derivatives were completely assigned based on one and homo- and heteronuclear two dimensional NMR experiments (COSY, HSQC, HMBC). The ^1^H-NMR spectra of compounds **5f**–**j** were recorded in dilute solution, as a pronounced decrease in solubility was observed in case of long chain derivatives. The isomeric ratio has also been determined by NMR in dilute solution at 35 °C, in order to exclude the misinterpretation originating from the different solubility of the isomers. According to earlier ^1^H spectroscopic studies of tartaric acid diamides, two set of signals were observed in case of the final products [15]. The small rate of isomerisation on the chiral centers of the (2*R*,3*R*)-tartaric moiety occurred only in the last step of the synthesis, resulted the two set of signals. The characteristic NMR peaks of (*R,R*) isomers were observed at 3.25 ppm (-CH_2_-NH-), 4.23 ppm (HO-CH-) and 5.50 ppm (-OH) respectively, while the (*RS*/*SR*) product exhibited signals at 3.29 ppm (-CH_2_-NH-), 4.00 ppm (HO-CH-) and 5.70 ppm (-OH). For the detailed ^1^H- and ^13^C-NMR assignments and MS data see the Experimental section. 

### 2.3. Proapoptotic effect of tartaric acid diamides

**Figure 2:**
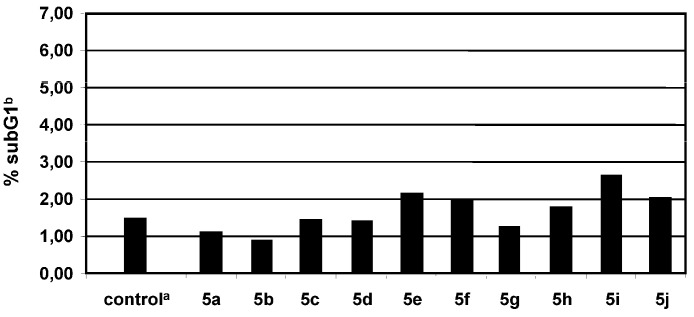
The pro-apoptotic effect of the newly synthesized compounds compared to pure solvent (compounds are considered pro-apoptotic over the rate of 20%).

As ceramides are key mediators in apoptosis [16], preliminary apoptotic studies were performed with compounds **5a**–**j** as follows: primer mouse embryonic fibroblasts were incubated with 10^−5^ mol/L of the compounds for 24 h and nucleosomal DNA fragmentation, a marker of apoptotic cell death, was analyzed by flow cytometry. Apoptotic cells are identified by their subdiploid DNA content after staining with the DNA intercalating dye, propidium iodide [17,18,19]. The percents of apoptotic cells corresponding to the sub-G_1_ phase were found to be not significantly different from those prepared with pure solvent that proved the absence of ceramide-like pro-apoptotic activity, which is an advantage for transdermal delivery systems (Figure 2). Compounds **5a**–**j** were also tested in the PAMPA (Parallel Artificial Membrane Permeability Assay) *stratum corneum* lipid matrix model [20].

## 3. Experimental Section

### 3.1. General

All the chemicals were from Sigma-Aldrich. Melting points of compounds were determined on a Stuart Scientific Digital Melting Point Apparatus SMP3. Mass spectra were obtained on a Agilent Technologies 6410 Triplequad LC/MS instrument. ^1^H-NMR and ^13^C-NMR were recorded on a 600 MHz Varian VNMRJ spectrometer equipped with a dual 5-mm inverse-detection gradient (IDPFG) probehead using TMS as internal reference. Proapoptotic effect was measured on FACSCalibur (Becton Dickinson, San Jose, CA, USA) flow cytometer using the CellQuest 3.1 (Becton Dickinson) software.

### 3.2. General procedure for the synthesis of compounds ***2a–d***

(*R,R*)-4-(Acetyloxy)-2,5-dioxooxolan-3-yl acetate (**1**, 25 mmol) was reacted with appropriate amine (25 mmol) in THF (40 mL) at room temperature overnight. Small amounts of the ammonium salts of products could be isolated by filtration, were crystallized from the reaction mixture. Then THF was removed under reduced pressure giving the 2,3-bis(acetyloxy)-3-(alkylcarbamoyl)propanoic acids **2a**–**d**.

*2,3-bis(Acetyloxy)-3-(octylcarbamoyl)propanoic acid* (**2a**). Yield: 86%; pale yellow oil; ^1^H-NMR (CDCl_3_) δ: 6.35 (1H; t; NH; *J* = 6 Hz), 5.75 (1H; d; CH; *J* = 2.4 Hz), 5.57 (1H; d; CH; *J* = 2.4 Hz), 3.32 (1H; m; NH-CH_2_), 3.19 (1H; m; NH-CH_2_), 2.17 (3H; s; CH_3_-COO), 2.11 (3H; s; CH_3_-COO), 1.49 (2H; tt; NH-CH_2_-CH_2_; *J* = 7.2, 6.6 Hz), 1.31-1.19 (10H; m; CH_2_), 0.86 (3H; t; CH_3_; *J* = 7.2 Hz); isomer ratio (*RR*/*RS*:*SR*): 100:0.

*2,3-bis(Acetyloxy)-3-(dodecylcarbamoyl)propanoic acid* (**2b**). Yield: 80%; yellowish oil; ^1^H-NMR (CDCl_3_) δ: 6.25 (1H; t; NH; *J* = 6 Hz), 5.78 (1H; d; CH; *J* = 2.4 Hz), 5.61 (1H; d; CH; *J* = 2.4 Hz), 3.36 (1H; m; N-CH_2_), 3.23 (1H; m; N-CH_2_), 2.20 (3H; s; CH_3_-COO), 2.13 (3H; s; CH_3_-COO), 1.49 (2H; tt; NH-CH_2_-CH_2_; *J* = 7.2, 6.6 Hz), 1.34–1.20 (18H; m; CH_2_), 0.88 (3H; t; CH_3_; *J* = 6.9 Hz); isomer ratio (*RR*/*RS*:*SR*): 100:0.

*2,3-bis(Acetyloxy)-3-(hexadecylcarbamoyl)propanoic acid* (**2c**). Yield: 81%; m.p. 76–78 °C; ^1^H-NMR (MeOD) δ: 5.62 (1H; d; CH; *J* = 2.4 Hz), 5.60 (1H; d; CH; *J* = 2,4 Hz), 3.25 (1H; m; NH-CH_2_), 3.19 (1H; m; NH-CH_2_), 2.18 (3H; s; CH_3_-COO), 2.13 (3H; s; CH_3_-COO), 1.51 (2H; tt; NH-CH_2_-CH_2_; *J* = 7.2, 6.6 Hz), 1.35-1.22 (26H; m; CH_2_), 0.92 (3H; t; CH_3_; *J* = 7.2 Hz); isomer ratio (*RR*/*RS*:*SR*): 100:0.

*2,3-bis(Acetyloxy)-3-(octadecylcarbamoyl)propanoic acid* (**2d**). Yield: 60%; m.p. 127–129 °C; ^1^H-NMR (CDCl_3_) δ: 6.25 (1H; t; NH; *J* = 6 Hz), 5.78 (1H; d; CH; *J* = 2.4 Hz), 5.62 (1H; d; CH; *J* = 2,4 Hz), 3.36 (1H; m; NH-CH_2_), 3.23 (1H; m; NH-CH_2_), 2.20 (3H; s; CH_3_-COO), 2.14 (3H; s; CH_3_-COO), 1.52 (2H; tt; NH-CH_2_-CH_2_; *J* = 6.6, 6.0 Hz), 1.38–1.20 (30H; m; CH_2_), 0.88 (3H; t; CH_3_; *J* = 7.2 Hz); isomer ratio (*RR*/*RS*:*SR*): 100:0.

### 3.3. General procedure for the synthesis of compounds ***3a–d***

*2,3-bis(Acetyloxy)-3-(alkylcarbamoyl)propanoic acid* **2a**–**d** (19 mmol) was treated with thionyl chloride (45 mL) and pyridine (0.7 mL) at 70 °C for 15 min. After evaporating the thionyl chloride, the 4-(acetyloxy)-1-alkyl-2,5-dioxopyrrolidin-3-yl acetate **3a**–**d** was purified on activated carbon in dichloromethane (15 mL). After evaporation of dichloromethane the product appears as a semisolid, which was used directly in the next step.

*4-(Acetyloxy)-1-octyl-2,5-dioxopyrrolidin-3-yl acetate* (**3a**). Yield: 78%; semisolid wax; ^1^H-NMR (DMSO-d_6_) δ: 5.77 (2H; s; CH), 3.42 (1H; m; N-CH_2_), 3.39 (1H; m; N-CH_2_), 2.13 (6H; s; CH_3_-COO), 1.47 (2H; tt; NH-CH_2_-CH_2_; *J* = 6.6, 6.0 Hz), 1.27-1.16 (10H; m; CH_2_), 0.84 (3H; t; CH_3_; *J* = 6.9 Hz); isomer ratio (*RR*/*RS*:*SR*): 100:0.

*4-(Acetyloxy)-1-dodecyl-2,5-dioxopyrrolidin-3-yl acetate* (**3b**). Yield: 85%; semisolid wax; ^1^H-NMR (DMSO-d_6_) δ: 5.77 (2H; s; CH), 3.43 (1H; m; N-CH_2_), 3.39 (1H; m; N-CH_2_), 2.14 (6H; s; CH_3_-COO), 1.49 (2H; tt; NH-CH_2_-CH_2_; *J* = 6.6, 6.0 Hz), 1.28–1.18 (18H; m; CH_2_), 0.85 (3H; t; CH_3_; *J* = 6.9 Hz); isomer ratio (*RR*/*RS*:*SR*): 100:0.

*4-(Acetyloxy)-1-hexadecyl-2,5-dioxopyrrolidin-3-yl acetate* (**3c**). Yield: 82%; brownish semisolid wax; ^1^H-NMR (DMSO-d_6_) δ: 5.76 (2H; s; CH), 3.43 (1H; m; N-CH_2_), 3.39 (1H; m; N-CH_2_), 2.14 (6H; s; CH_3_-COO), 1.49 (2H; tt; NH-CH_2_-CH_2_; *J* = 6.6, 6.0 Hz), 1.29–1.18 (26H; m; CH_2_), 0.85 (3H; t; CH_3_; *J* = 6.9 Hz); isomer ratio (*RR*/*RS*:*SR*): 100:0.

*4-(Acetyloxy)-1-octadecyl-2,5-dioxopyrrolidin-3-yl acetate* (**3d**). Yield: 79%; brownish semisolid wax; ^1^H-NMR (DMSO-d_6_) δ: 5.76 (2H; s; CH), 3.42 (1H; m; N-CH_2_), 3.38 (1H; m; N-CH_2_), 2.15 (6H; s; CH_3_-COO), 1.50 (2H; tt; NH-CH_2_-CH_2_; *J* = 6.6, 6.0 Hz), 1.31–1.20 (30H; m; CH_2_), 0.84 (3H; t; CH_3_; *J* = 7.2 Hz); isomer ratio (*RR*/*RS*:*SR*): 100:0.

### 3.4. General procedure for the synthesis of compounds ***4a***–***d***

The 4-(acetyloxy)-1-alkyl-2,5-dioxopyrrolidin-3-yl acetates **3a**–**d** (15 mmol) were deacetylated by dissolving them in ethanol (90 mL) and slowly adding acetyl chloride (20 mL) at 0 °C, then stirring the mixture for 1 day at room temperature. The precipitated white crystalline 3,4-dihydroxy-1-alkyl-pyrrolidine-2,5-dione products **4a**–**d** were filtered and washed with ethanol.

*3,4-Dihydroxy-1-octylpyrrolidine-2,5-dione* (**4a**). Yield: 76%; m.p. 127–129 °C; ^1^H-NMR (CDCl_3_) δ: 4.57 (2H; s; CH), 3.54 (1H; m; NH-CH_2_), 3.50 (1H; m; NH-CH_2_), 1.56 (2H; m; NH-CH_2_-CH_2_), 1.32–1.22 (10H; m; CH_2_), 0.88 (3H; t; CH_3_; *J* = 7.2 Hz); isomer ratio (*RR*/*RS*:*SR*): 100:0.

*1-Dodecyl-3,4-dihydroxypyrrolidine-2,5-dione* (**4b**). Yield: 83%; m.p. 142–144 °C; ^1^H-NMR (CDCl_3_) δ: 4.58 (2H; s; CH), 3.55 (1H; m; NH-CH_2_), 3.50 (1H; m; NH-CH_2_), 1.58 (2H; m; NH-CH_2_-CH_2_), 1.36–1.21 (18H; m; CH_2_), 0.88 (3H; t; CH_3_; *J* = 7.2 Hz); isomer ratio (*RR*/*RS*:*SR*): 100:0.

*1-Hexadecyl-3,4-dihydroxypyrrolidine-2,5-dione* (**4c**). Yield: 81%; m.p. 140–142 °C; ^1^H-NMR (DMSO-d_6_) δ: 4.32 (2H; s; CH), 3.54 (1H; m; NH-CH_2_), 3.50 (1H; m; NH-CH_2_), 1.56 (2H; m; NH-CH_2_-CH_2_), 1.32-1.22 (26H; m; CH_2_), 0.88 (3H; t; CH_3_; *J* = 7.2 Hz); isomer ratio (*RR*/*RS*:*SR*): 100:0.

*3,4-Dihydroxy-1-octadecylpyrrolidine-2,5-dione* (**4d**). Yield: 75%; m.p. 132–134 °C; ^1^H-NMR (CDCl_3_) δ: 4.57 (2H; s; CH), 3.54 (1H; m; NH-CH_2_), 3.50 (1H; m; NH-CH_2_), 1.56 (2H; m; NH-CH_2_-CH_2_), 1.32-1.21 (30H; m; CH_2_), 0.88 (3H; t; CH_3_; *J* = 7.2 Hz); isomer ratio (*RR*/*RS*:*SR*): 100:0.

### 3.5. General procedure for the synthesis of compounds ***5a***–***j***

3,4-Dihydroxy-1-alkylpyrrolidine-2,5-dione **4a**–**d** (4 mmol) was treated with appropriate amine (8 mmol) in xylene (50 mL) at 130 °C for 24 h. The white crystalline 2,3-dihydroxy-*N,N′*-dialkyl-butanediamide product **5a**–**j** was filtered and washed with hot ethanol.

*2,3-Dihydroxy-N,N′-dioctylbutanediamide* (**5a**). Yield: 79%; m.p. 172–174 °C; ^1^H-NMR (CDCl_3_) δ: 7.08 (2H; t; NH; *J* = 6 Hz), 5.70 (0.28H; s; OH), 5.50 (1.72H; d; OH; *J* = 6.6 Hz), 4.23 (1.72H; d; CH; *J* = 6.6 Hz), 4.00 (0.28H; s; CH), 3.29 (0.56H; m; NH-CH_2_), 3.24 (3.44H; m; NH-CH_2_), 1.50 (4H; tt; NH-CH_2_-CH_2_; *J* = 7.2, 6.6 Hz), 1.37–1.22 (20H; m; CH_2_), 0.88 (6H; t; CH_3_; *J* = 7.0 Hz); ^13^C-NMR (CDCl_3_) δ: 174.6, 70.8, 39.8, 32.5, 30.0, 29.9, 29.8, 27.4, 23.3, 14.7; isomer ratio (*RR*/*RS*:*SR*): 86:14; MS (ESI) m/z: 373 (MH^+^).

*N-Dodecyl-2,3-dihydroxy-N′-octylbutanediamide* (**5b**). Yield: 75%; m.p. 166–168 °C; ^1^H-NMR (CDCl_3_) δ: 7.08 (2H; t; NH; *J* = 6 Hz), 5.70 (0.22H; s; OH), 5.50 (1.78H; d; OH; *J* = 6.6 Hz), 4.23 (1.78H; d; CH; *J* = 6.6 Hz), 4.00 (0.22H; s; CH), 3.29 (0.44H; m; NH-CH_2_), 3.24 (3.56H; m; NH-CH_2_), 1.50 (4H; tt; NH-CH_2_-CH_2_; *J* = 7.2, 6.6 Hz), 1.37-1.22 (28H; m; CH_2_), 0.88 (6H; t; CH_3_; *J* = 6.9 Hz); ^13^C-NMR (CDCl_3_) δ: 174.6, 70.6, 39.8, 32.6, 30.34, 30.31, 30.28, 30.21, 30.04, 29.9, 29.8, 27.4, 23.4, 14.8; isomer ratio (*RR*/*RS*:*SR*): 89:11; MS (ESI) m/z: 429 (MH^+^).

*N-Hexadecyl-2,3-dihydroxy-N′-octylbutanediamide* (**5c**). Yield: 93%; m.p. 161–163 °C; ^1^H-NMR (CDCl_3_) δ: 7.08 (2H; t; NH; *J* = 6 Hz), 5.70 (0.40H; s; OH), 5.50 (1.60H; d; OH; *J* = 6.6 Hz), 4.23 (1.60H; d; CH; *J* = 6.6 Hz), 4.00 (0.40H; s; CH), 3.29 (0.80H; m; NH-CH_2_), 3.24 (3.20H; m; NH-CH_2_), 1.50 (4H; tt; NH-CH_2_-CH_2_; *J* = 7.2, 6.6 Hz), 1.38-1.22 (36H; m; CH_2_), 0.88 (6H; t; CH_3_; *J* = 6.7 Hz); ^13^C-NMR (CDCl_3_) δ: 174.6, 70.6, 39.8, 32.6, 30.38, 30.34, 30.31, 30.28, 30.21, 30.11, 30.04, 30.00, 29.96, 29.93, 29.86, 27.4, 23.4, 14.8; isomer ratio (*RR*/*RS*:*SR*): 80:20; MS (ESI) m/z: 485 (MH^+^).

*2,3-Dihydroxy-N-octadecyl-N′-octylbutanediamide* (**5d**). Yield: 92%; m.p. 163–165 °C; ^1^H-NMR (CDCl_3_) δ: 7.08 (2H; t; NH; *J* = 6 Hz), 5.70 (0.36H; s; OH), 5.50 (1.64H; d; OH; *J* = 6.6 Hz), 4.23 (1.64H; d; CH; *J* = 6.6 Hz), 4.00 (0.36H; s; CH), 3.29 (0.72H; m; NH-CH_2_), 3.24 (3.28H; m; NH-CH_2_), 1.50 (4H; tt; NH-CH_2_-CH_2_; *J* = 7.2, 6.6 Hz), 1.38-1.22 (40H; m; CH_2_), 0.88 (6H; t; CH_3_; *J* = 6.7 Hz); ^13^C-NMR (CDCl_3_) δ: 174.6, 70.6, 39.8, 32.6, 30.39, 30.35, 30.33, 30.29, 30.26, 30.22, 30.16, 30.11, 30.07, 30.04, 29.99, 29.93, 29.86, 27.4, 23.4, 14.8; isomer ratio (*RR*/*RS*:*SR*): 82:18; MS (ESI) m/z: 513 (MH^+^).

*N,N′-Didodecyl-2,3-dihydroxybutanediamide* (**5e**). Yield: 86%; m.p. 155–157 °C; ^1^H-NMR (CDCl_3_) δ: 7.08 (2H; t; NH; *J* = 6 Hz), 5.70 (0.28H; s; OH), 5.50 (1.72H; d; OH; *J* = 6.6 Hz), 4.23 (1.72H; d; CH; *J* = 6.6 Hz), 4.00 (0.28H; s; CH), 3.29 (0.56H; m; NH-CH_2_), 3.24 (3.44H; m; NH-CH_2_), 1.50 (4H; tt; NH-CH_2_-CH_2_; *J* = 7.2, 6.6 Hz), 1.38-1.22 (36H; m; CH_2_), 0.88 (6H; t; CH_3_; *J* = 6.9 Hz); ^13^C-NMR (CDCl_3_) δ: 173.6, 70.8, 39.8, 32.6, 30.32, 30.28, 30.24, 30.21, 30.14, 30.04, 29.92, 27.5, 23.4, 14.8; isomer ratio (*RR*/*RS*:*SR*): 86:14; MS (ESI) m/z: 485 (MH^+^).

*N-Dodecyl-N′-hexadecyl-2,3-dihydroxybutanediamide* (**5f**). Yield: 86%; m.p. 157–159 °C; ^1^H-NMR (CDCl_3_) δ: 7.08 (2H; t; NH; *J* = 6 Hz), 5.70 (0.38H; br s; OH), 5.50 (1.62H; br s; OH), 4.23 (1.62H; br s; CH), 4.00 (0.38H; br s; CH), 3.29 (0.76H; m; NH-CH_2_), 3.24 (3.24H; m; NH-CH_2_), 1.50 (4H; tt; NH-CH_2_-CH_2_; *J* = 7.2, 6.6 Hz), 1.38-1.22 (44H; m; CH_2_), 0.88 (6H; t; CH_3_; *J* = 6.7 Hz); ^13^C-NMR (CDCl_3_) δ: 173.5, 70.6, 39.8, 32.6, 30.39, 30.35, 30.32, 30.29, 30.25, 30.23, 30.21, 30.19, 30.04, 30.00, 29.91, 27.5, 23.4, 14.8; isomer ratio (*RR*/*RS*:*SR*): 81:19; MS (ESI) m/z: 541 (MH^+^).

*N-Dodecyl-2,3-dihydroxy-N′-octadecylbutanediamide* (**5g**). Yield: 56%; m.p. 151–154 °C; ^1^H-NMR (CDCl_3_) δ: 7.08 (2H; t; NH; *J* = 6 Hz), 5.70 (0.46H; s; OH), 5.50 (1.54H; d; OH; *J* = 6.6 Hz), 4.23 (1.54H; d; CH; *J* = 6.6 Hz), 4.00 (0.46H; s; CH), 3.29 (0.92H; m; NH-CH_2_), 3.24 (3.08H; m; NH-CH_2_), 1.50 (4H; tt; NH-CH_2_-CH_2_; *J* = 7.2, 6.6 Hz), 1.38–1.22 (48H; m; CH_2_), 0.88 (6H; t; CH_3_; *J* = 6.7 Hz); ^13^C-NMR (CDCl_3_) δ: 173.6, 70.6, 39.8, 32.6, 30.39, 30.35, 30.32, 30.29, 30.27, 30.24, 30.22, 30.20, 30.17, 30.14, 30.10, 30.04, 29.93, 27.5, 23.4, 14.8; isomer ratio (*RR*/*RS*:*SR*): 77:23; MS (ESI) m/z: 569 (MH^+^).

*N,N′-Dihexadecyl-2,3-dihydroxybutanediamide* (**5h**). Yield: 96%; m.p. 157–159 °C; ^1^H-NMR (CDCl_3_) δ: 7.08 (2H; t; NH; *J* = 6 Hz), 5.70 (0.34H; s; OH), 5.50 (1.66H; d; OH; *J* = 6.6 Hz), 4.23 (1.66H; d; CH; *J* = 6.6 Hz), 4.00 (0.34H; s; CH), 3.29 (0.68H; m; NH-CH_2_), 3.24 (3.32H; m; NH-CH_2_), 1.50 (4H; tt; NH-CH_2_-CH_2_; *J* = 7.2, 6.6 Hz), 1.38–1.22 (52H; m; CH_2_), 0.88 (6H; t; CH_3_; *J* = 6.8 Hz); ^13^C-NMR (CDCl_3_) δ: 173.6, 70.8, 39.8, 32.6, 30.39, 30.37, 30.35, 30.32, 30.29, 30.26, 30.22, 30.19, 30.05, 30.01, 29.92, 27.5, 23.3, 14.8; isomer ratio (*RR*/*RS*:*SR*): 86:14; MS (ESI) m/z: 597 (MH^+^).

*N-Hexadecyl-2,3-dihydroxy-N′-octadecylbutanediamide* (**5i**). Yield: 82%; m.p. 151–153 °C; ^1^H-NMR (CDCl_3_) δ: 7.08 (2H; t; NH; *J* = 6 Hz), 5.70 (0.40H; s; OH), 5.50 (1.60H; d; OH; *J* = 6.6 Hz), 4.23 (1.60H; d; CH; *J* = 6.6 Hz), 4.00 (0.40H; s; CH), 3.29 (0.80H; m; NH-CH_2_), 3.24 (3.20H; m; NH-CH_2_), 1.50 (4H; tt; NH-CH_2_-CH_2_; *J* = 7.2, 6.6 Hz), 1.38-1.22 (56H; m; CH_2_), 0.88 (6H; t; CH_3_; *J* = 6.8 Hz); ^13^C-NMR (CDCl_3_) δ: 173.6, 70.8, 39.8, 32.6, 30.39, 30.37, 30.35, 30.31, 30.26, 30.22, 30.19, 30.15, 30.12, 30.08, 30.05, 30.01, 29.92, 27.5, 23.4, 14.8; isomer ratio (*RR*/*RS*:*SR*): 80:20; MS (ESI) m/z: 625 (MH^+^).

*2,3-Dihydroxy-N,N′-dioctadecylbutanediamide* (**5j**). Yield: 91%; m.p. 151–153 °C; ^1^H-NMR (CDCl_3_) δ: 7.08 (2H; t; NH; *J* = 6 Hz), 5.70 (0.60H; s; OH), 5.50 (1.40H; d; OH; *J* = 6.6 Hz), 4.23 (1.40H; d; CH; *J* = 6.6 Hz), 4.00 (0.60H; s; CH), 3.29 (1.20H; m; NH-CH_2_), 3.24 (2.80H; m; NH-CH_2_), 1.50 (4H; tt; NH-CH_2_-CH_2_; *J* = 7.2, 6.6 Hz), 1.38–1.22 (60H; m; CH_2_), 0.88 (6H; t; CH_3_; *J* = 6.9 Hz); ^13^C-NMR (CDCl_3_) δ: 173.4, 70.9, 39.8, 32.6, 30.38, 30.35, 30.31, 30.29, 30.26, 30.23, 30.19, 30.15, 30.10, 30.07, 30.05, 30.00, 29.92, 27.5, 23.3, 14.8; isomer ratio (*RR*/*RS*:*SR*): 70:30; MS (ESI) m/z: 653 (MH^+^).

## 4. Conclusions

In this study long aliphatic chain tartramides which are able to replace ceramides in *in vitro* model of *stratum corneum* lipid matrix were synthesized. It can also be concluded that the ceramide-like tartramides, if applied as host penetration enhancer compounds and based on their proapotopic effects, exhibit high degrees of safety, and their inclusion enables the gradual release of medicines, agricultural chemicals and perfumes and the reduction of the emergent toxicity thereof. Therefore, it can be supposed that the long chain tartaric acid amide derivatives have very meaningful future in the medicinal chemistry.
